# The eukaryotic linear motif resource – 2018 update

**DOI:** 10.1093/nar/gkx1077

**Published:** 2017-11-10

**Authors:** Marc Gouw, Sushama Michael, Hugo Sámano-Sánchez, Manjeet Kumar, András Zeke, Benjamin Lang, Benoit Bely, Lucía B Chemes, Norman E Davey, Ziqi Deng, Francesca Diella, Clara-Marie Gürth, Ann-Kathrin Huber, Stefan Kleinsorg, Lara S Schlegel, Nicolás Palopoli, Kim V Roey, Brigitte Altenberg, Attila Reményi, Holger Dinkel, Toby J Gibson

**Affiliations:** Structural and Computational Biology Unit, European Molecular Biology Laboratory, Heidelberg 69117, Germany; Institute of Enzymology, Research Centre for Natural Sciences, Hungarian Academy of Sciences, Budapest H-1117, Hungary; European Molecular Biology Laboratory, European Bioinformatics Institute (EMBL-EBI), Wellcome Trust Genome Campus, Hinxton, Cambridge CB10 1SD, UK; Protein Structure-Function and Engineering Laboratory, Fundación Instituto Leloir and IIBBA-CONICET, Buenos Aires CP 1405, Argentina; Departamento de Fisiología y Biología Molecular y Celular, Facultad de Ciencias Exactas y Naturales, Universidad de Buenos Aires, Buenos Aires CP 2160, Argentina; Instituto de Investigaciones Biotecnoltógicas, Universidad Nacional de General San Martín, IIB-INTECH-CONICET, San Martín, Buenos Aires CP 1650, Argentina; UCD School of Medicine & Medical Science, University College Dublin, Belfield, Dublin 4, Ireland; Ruprecht-Karls-Universität, Heidelberg 69117, Germany; Department of Science and Technology, Universidad Nacional de Quilmes, CONICET, Bernal B1876BXD, Buenos Aires, Argentina; Leibniz-Institute on Aging, Fritz Lipmann Institute (FLI), Jena D-07745, Germany

## Abstract

Short linear motifs (SLiMs) are protein binding modules that play major roles in almost all cellular processes. SLiMs are short, often highly degenerate, difficult to characterize and hard to detect. The eukaryotic linear motif (ELM) resource (elm.eu.org) is dedicated to SLiMs, consisting of a manually curated database of over 275 motif classes and over 3000 motif instances, and a pipeline to discover candidate SLiMs in protein sequences. For 15 years, ELM has been one of the major resources for motif research. In this database update, we present the latest additions to the database including 32 new motif classes, and new features including Uniprot and Reactome integration. Finally, to help provide cellular context, we present some biological insights about SLiMs in the cell cycle, as targets for bacterial pathogenicity and their functionality in the human kinome.

## INTRODUCTION

Short linear motifs (SLiMs) are small functional protein modules that mediate protein–protein interactions and protein sequence modifications (1,2). They play essential roles in almost all cellular processes, including cell signaling, trafficking, protein stability, cell-cycle progression and molecular switching mechanisms (2–5). SLiMs have also been found to play an increasingly important role in human disease, including viral pathogenicity (6) and are also emerging as major players in cancer, especially the degron class of motifs (7,8).

SLiMs are short degenerate sequences, generally between 3 and 15 amino acids in length, and are typically formed by a few highly conserved residues located between more loosely conserved positions (1). As a result, an individual motif binds with relatively weak affinity, usually in the low micromolar range. However, multiple SLiMs often cooperate to create strong yet dynamic interfaces. They generally occur in intrinsically disordered regions, and (in the absence of a binding partner) have no stable three dimensional structure. Although SLiMs are short and mostly participate in transient interactions, they are essential to a protein’s binding specificity and proper functioning. Current estimates suggest there may be in the order of 1 000 000 different SLiMs in the human proteome (9). However, despite their abundance and importance, far fewer have been properly described. The eukaryotic linear motif (ELM) resource is a project dedicated to cataloging, characterizing and identifying these motifs.

## THE ELM RESOURCE

The ELM resource is a database and web server focused on SLiMs (elm.eu.org). ELM was first released in 2003 (10), and has grown into one of the most widely used and reliable resources for high quality SLiM annotations, mostly focused on, but not limited to, eukaryotic proteins (11–13). The resource consists of two main components: a manually curated database of SLiM definitions and an exploratory pipeline which uses these definitions to look for putative SLiMs in protein sequences.

The database component of ELM contains manually curated characterizations of over 275 SLiMs contributed by our community of biologists and biocurators. Each SLiM (named ‘motif class’ in ELM) is defined using a regular expression, a computational syntax that can express complex patterns of letters (or single letter amino acid abbreviations). For example, the regular expression ...[ST]P[RK] is used to express an amino acid sequence that starts with any three amino acids ... before either a serine S or threonine T, followed by a proline P, and ending in an arginine R or lysine K. Curators annotate each motif class based on experimentally validated motif occurrences (named ‘motif instances’ in ELM) from the scientific literature. Each motif class annotation is accompanied by a detailed description, links to the original studies and crosslinks to external databases and ontologies including the Gene Ontology (14), Proteomics standards initiative–molecular interaction (PSI–MI; (15)), the NCBI taxonomy (16,17) and the Protein Data Bank (PDB; (18)).

The ELM exploration pipeline is used to detect matches to SLiMs in protein sequences. When a user submits a sequence, it is matched against all regular expressions annotated in the ELM database. Since SLiM patterns are short and often highly degenerate, SLiM pattern matching alone is likely to generate many false positive predictions. Any motifs likely to be non-functional are deprecated by applying structure and domain architecture filters based on protein disorder (from GlobPlot (19) and IUPred (20)), protein secondary structure (21) and protein domains (from SMART (22) and Pfam (23)). The result contains putative SLiMs located in disordered regions that are accessible for making binding interactions. The motif occurrences are also given a conservation score to reflect how conserved this sequence is across aligned homologous proteins. The results of the ELM exploration pipeline are a useful starting point for inferring possible functions of a protein and selecting novel candidates for further examination with other bioinformatics resources. For example, the context of motifs in a sequence alignment and other information such as intrinsic disorder prediction and disease mutations can be visualized with ProViz (24). To follow up interesting individual motifs a tool such as SLiMSearch (25) can query protein databases, providing a ranking for your protein of interest relative to other proteins containing the motif.

## NEW CONTENT IN ELM

### New ELM classes

**Figure 1.**
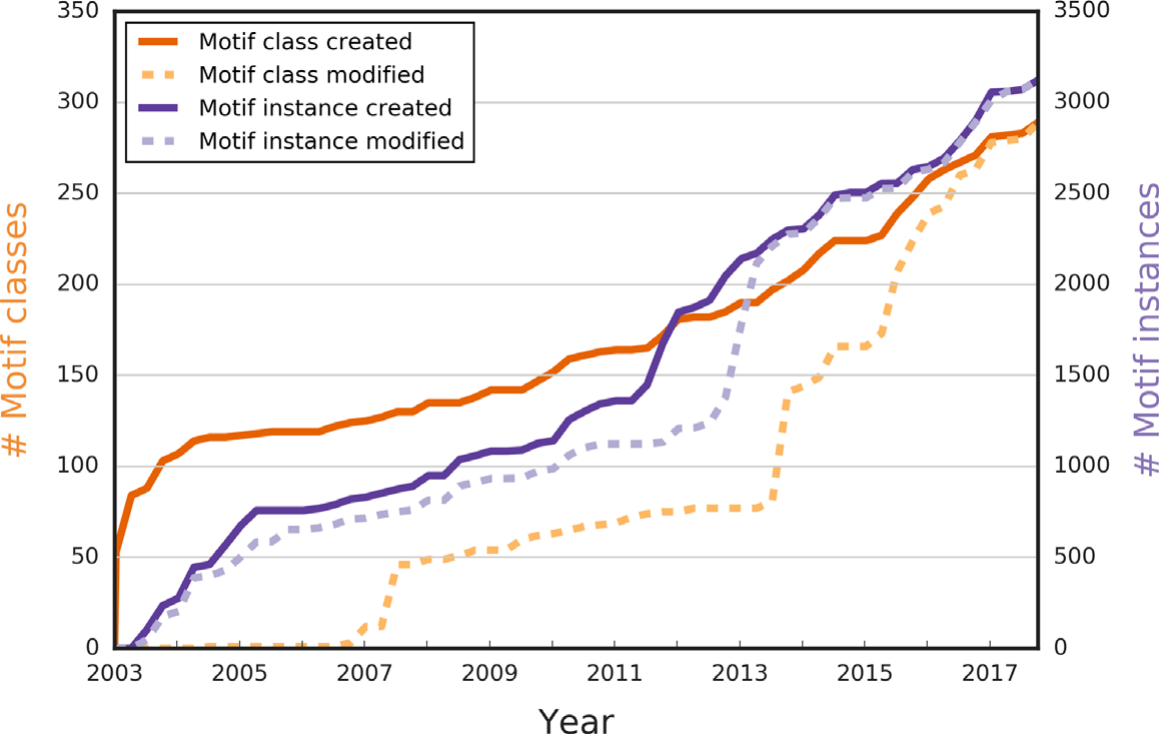
The number of SLiMs (motif classes and motif instances) created and modified in ELM. For the past 15 years, ELM has been steadily growing, and the total number of motif classes (dark orange) and motif instances (dark purple) continues to grow each year. As of September 2017, there are over 275 motif classes defined, and over 3000 motif instances annotated. Besides contributing new content, curators also updated existing annotations to include new findings (last modification dates for motif classes in dashed light purple and for motif instances in dashed light orange).

The main type of data curated in ELM are the motif classes. Each motif class consists of the SLiM name and description, its regular expression, and the complete set of motif instances and experimental data used to define the class. Currently there are over 275 motif classes, 32 more than in the last *NAR* publication in 2016 (13) (Figure 1 and Table 1). Most notably six variants of the mitogen-activated protein kinases (MAPK) docking D-motifs and additions and improvements to cell cycle regulatory motifs including relevant degrons and kinases such as the Polo-like kinases (Plks). We have tried to be comprehensive for degron motifs (recently reviewed in (8)) with the most recent addition being the pLxIS motif involved in immune response of interferon-regulatory factor IRF3 but which has degron-like properties for rotavirus hijacking (26). Another example of a hijacked motif is the tyrosine-kinase regulating motif EPIYA, which is a common motif mimic used by pathogenic bacteria. Also, several existing motif entries have been redefined or expanded, including recent updates to the abundant and versatile class of 14-3-3-binding motifs and to the cell cycle checkpoint retinoblastoma protein pRb-binding LxCxE motif.

**Table 1.** New ELM motif classes

ELM motif class identifier	# Instances	ELM motif class description
DEG_COP1_1	12	A destruction motif interacts with the COP1 WD 40 domain for target ubiquitination and degradation.
DOC_MAPK_DCC_7	11	A kinase docking motif mediating interaction toward the ERK1/2 and p38 subfamilies of MAP kinases.
DOC_MAPK_HePTP_8	10	A kinase docking motif that interacts with the ERK1/2 and p38 subfamilies of MAP kinases.
DOC_MAPK_JIP1_4	29	A shorter D site specifically recognized by the JNK kinases
DOC_MAPK_MEF2A_6	24	A kinase docking motif that mediates interaction toward the ERK1/2 and p38 subfamilies of MAP kinases.
DOC_MAPK_NFAT4_5	17	An extended D site specifically recognized by the JNK kinases.
DOC_MAPK_RevD_3	6	Reverse (C to N direction) of the classical MAPK docking motif ELM:DOC_MAPK_gen_1 with an often extended linker region of the bipartite motif.
DOC_PP2A_B56_1	18	Docking site required for the regulatory subunit B56 of PP2A for protein dephosphorylation.
LIG_14-3-3_CanoR_1	62	Canonical Arg-containing phospho-motif mediating a strong interaction with 14-3-3 proteins.
LIG_14-3-3_ChREBP_3	1	14-3-3 protein binding to a nonphosphorylated helical peptide in ChREBP is promoted by adenosine monophosphate.
LIG_14-3-3_CterR_2	5	C-terminal Arg-containing phospho-motif mediating a strong interaction with 14-3-3 proteins.
LIG_ANK_PxLPxL_1	10	The consensus PxLPxI/L motif, which can be found in diverse proteins, binds to the ankyrin repeat domains of ANKRA2 and its close paralog RFXANK.
LIG_APCC_ABBA_1	11	Amphipathic motif that is involved in APC/C inhibition by binding of CDH1/CDC20. In metazoan cyclin A, the motif also acts as a degron, enabling the cyclin’s degradation in prometaphase.
LIG_APCC_ABBAyCdc20_2	2	Amphipathic motif that binds to yeast Cdc20 and acts as an APC/C degron enabling cyclin Clb5 degradation during mitosis.
LIG_BH_BH3_1	19	The BH3 motif is found in pro-apoptotic proteins and interacts with BH domains of the anti-apoptotic Bcl-2 family members to regulate apoptosis.
LIG_CSK_EPIYA_1	13	Csk Src Homology 2 domain-binding EPIYA motif.
LIG_CSL_BTD_1	18	The motif mediates the interaction between a notch-like protein and the transcription factor CSL by placing two amino acids (W and P) into a hydrophobic pocket of the Beta-trefoil DNA-binding (BTD) domain of CSL.
LIG_G3BP_FGDF_1	9	The FGDF motif binds to a hydrophobic binding cleft within the N-terminal NTF2-like domain of the stress granule protein G3BP.
LIG_GBD_Chelix_1	12	Amphipatic α-helix that binds the GTPase-binding domain (GBD) in WASP and N-WASP.
LIG_GSK3_LRP6_1	8	PPPSP motif present on the cytosolic tails of the transmembrane receptors LRP5 and LRP6, responsible for GSK3 binding and inhibition when phosphorylated.
LIG_IRF3_LxIS_1	5	A binding site for IRF-3 protein present in various innate adaptor proteins and the viral protein NSP1to trigger the innate immune responsive pathways.
LIG_KLC1_WD_1	22	This short WD or WE motif is found in cargo proteins and mediates kinesin-1-dependent microtubule transport by binding to the KLC TPR region.
LIG_LRP6_Inhibitor_1	3	Short motif present in extracellular of some Wnt antagonists recognized by the N-terminal β-propeller domain of LRP5/6 and thus inhibits the Wnt pathway.
LIG_PALB2_WD40_1	1	A motif present in the BRCA2 protein which binds to the WD 40 repeat (blade 4,5) domain of PALB2 which is required for the recognition of DNA double strand breaks and repair.
LIG_Rrp6Rrp47_Mtr4_1	6	The motif enables the interaction of Mtr4 like helicaes with the Rrp6-Rrp47 heterodimer and thus the formation of the exosome-binding complex.
LIG_UFM1_UFIM_1	1	UFIM is a motif present in the E1 enzyme UBA5 required to bind ubiquitin-like protein UFM1. UFIM overlaps with a LIR motif binding LC3/GABARAP family proteins.
LIG_Vh1_VBS_1	12	An amphipathic α-helix recognized by the head domain of vinculin that is required for vinculin activation and actin filament attachment.
MOD_CDK_SPK_2	18	Short version of the cyclin-dependent kinases (CDK) phosphorylation site which shows specificity toward a lysine/arginine residue at the [ST] +2 position.
MOD_CDK_SPxxK_3	25	Longer version of the CDK phosphorylation site which shows specificity toward a lysine/arginine residue at position +4 after the phospho-Ser/Thr.
MOD_Plk_1	23	Ser/Thr residue phosphorylated by the Plk1 kinase.
MOD_Plk_2-3	3	Ser/Thr residue phosphorylated by Plk2 and Plk3.
MOD_Plk_4	7	Ser/Thr residue phosphorylated by Plk4.

Since the last NAR database issue publication 32 motif classes have been annotated to the database. (13)

### New ELM instances

One of the principal types of data contained in ELM are the motif instances, i.e. experimentally validated occurrences of motif classes in proteins. As of September 2017, ELM has 3093 instances, having added 491 new motif instances since the last *NAR* database publication (13) and also updated many existing entries (Figure 1). Following previous years, the majority of new motif instances are for human proteins and other animals, although we have had a large increase in the number of viral motif mimics and we have begun the process of adding instances of bacterial motif mimicry from a systematic review of the literature (Table 2).

**Table 2.** New ELM motif classes and instances

Motif type	Motif classes added	Motif classes modified	Taxon	Motif instances added	Motif instances modified
DEG	1	1	Human	315	10
CLV	0	1	other Animal	87	2
TRG	0	0	Fungi	17	0
LIG	19	9	Plant	10	3
MOD	5	2	Bacteria	23	0
DOC	7	5	Virus	39	0

Since the last NAR database issue publication in 2016 (13) a total of 32 motif classes and 491 motif instances have been added to the database. Most of the new motifs added are either Ligand (LIG) or Docking (DOC) motifs. Most of the new motif instances are Human, although motif instances for many other branches of life have also been added.

## NEW FEATURES IN ELM

In this release, we have further integrated ELM with other bioinformatics databases and resources. An important development is that UniProt (27) now includes ELM as a database cross-reference in the ‘protein–protein interaction databases’ section. We have also updated the experimental evidence codes used in ELM to the latest version of PSI–MI version 2.5 (15). The most notable changes in PSI–MI are that terms ‘GST-pulldown’ and ‘HIS-pulldown’ have each been demerged into a combination of terms: ‘glutathione s transferase tag’ and ‘pull down’ and ‘his tag’ and ‘pull down’. We have also integrated ELM with the Reactome pathway database (28), and introduced programmatic access to the ELM exploration pipeline, both of which we describe below in more detail.

### Reactome

One way to gain additional insights into which biological processes a SLiM may be involved in, is to examine the cellular pathways that contain proteins with this motif. ELM already has links to pathways contained in the KEGG pathway database (12,29). In order to augment the cellular network knowledge potential available in ELM, we have now integrated ELM with another pathway database: Reactome.

Reactome is a manually curated peer reviewed pathway database (28). Pathways are defined by reactions and the entities participating in them (nucleic acids, proteins, complexes and small molecules), and are supported by literature citations and expert curation. It is now possible to visualize and download all Reactome annotations for proteins available in ELM. Every protein in ELM having a Reactome annotation now has a link to display a Reactome pathway diagram that highlights where this protein functions. The complete list of Reactome annotations can also be retrieved from the ELM downloads page. Later in this paper, we will illustrate how the ELM annotated Reactome data can be used to analyze the motifs involved in the cell cycle.

### The ELM API

The ELM exploration pipeline is a useful tool to predict putative SLiMs in protein sequences. Nevertheless, the graphical user interface is not suitable for automated or large scale analyses. One of the latest updates to the ELM resource has been to include an application programmatic interface (API) to the ELM exploration pipeline (30). The ELM exploration API allows users to submit either a protein sequence or a UniProt ID to predict which SLiMs might exist in it. The protein sequence is matched against all of the regular expressions annotated in ELM and each motif match is passed through a combination of structural context filters, which help to predict whether the motif is likely to be biologically functional. Motif matches are filtered out of the predicted motifs if they occur in globular domains, transmembrane regions or extracellular regions. The API also returns whether any of the motifs detected are already annotated in ELM, or whether the motif has been annotated in a homolog in ELM. The output is provided as a *tsv* (tab separated values) file, which is easy to read and analyze computationally. The API can be accessed using any programming framework that can process HTTP requests, for example *wget, curl* and the python ‘requests’ package. For more information on using the API, usage guidelines and how to interpret the results, see (30) and read the documentation on elm.eu.org/api/manual.html.

## MOTIFS IN BACTERIAL PATHOGENS

Motifs are not unique to eukaryotes; they also exist in bacteria and viruses. It has been known for some time that viruses use motif mimicry to interfere with biological processes of the host cell (6). This behavior is not limited to viruses, but the data for pathogenic bacteria are more limited (31,32). In the latest version of ELM, we report instances from a handful of bacteria that are now known to use motif mimicry for pathogenicity.

Among the bacterial proteins with newly added motifs are OspF from *Shigella flexneri* and SpvC from *Salmonella Typhimurium*, which use a D-motif to recognize MAPK proteins like ERK, JNK or p38 and irreversibly modify a phosphorylated residue to block downstream MAPK signaling, thus preventing the activation of the immune response (33,34). Enterohaemorrhagic *Escherichia coli* uses the multi-valency of a GBD domain-binding motif to activate up to seven WASP proteins with a single effector protein, espFU (35,36). The same protein has five tandem PxxP motifs that bind to the SH3 domain of BAIAP2L1/IRTKS with the highest reported affinity for a motif-SH3 complex (500 *nM*) (35). Finally, the tyrosine-phosphorylated EPIYA motif present in the cellular protein Pragmin is also used by CagA from *Helicobacter pylori* and LspA1 from *Haemophilus ducreyi* to recruit CSK and phosphorylate Src-family kinases (37,38), interfering with cell fate and phagocytosis (39,40). Besides their role in pathogenicity, motif mimicry by bacteria also has implications for bacterial oncogenicity, such as the oncogenic potential of *H. pylori* strains (41).

## MOTIFS IN THE CELL CYCLE

**Figure 2.**
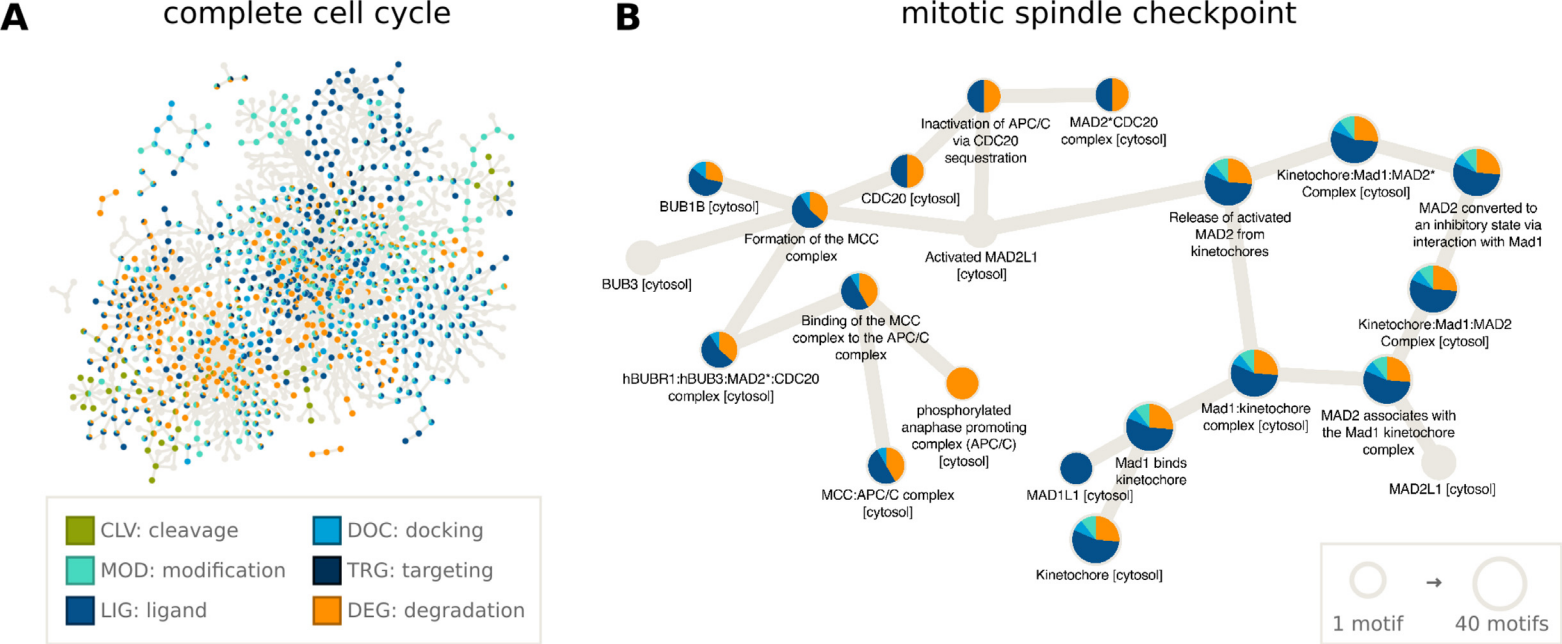
SLiMs play major roles in many biological pathways, including those involved in the progression of the cell cycle. Using the Reactome pathway annotations downloaded from ELM and using Cytoscape (42) to visualize the data, we can see that many SLiMs (especially Ligand and Degradation motifs) are involved in the cell cycle (**A**) and specifically in the mitotic spindle checkpoint (**B**).

One of the new features included in ELM are the protein’s pathway annotations from the Reactome database (28), which can be downloaded from the ELM ‘downloads’ page. These annotations allow the construction of network diagrams to examine the roles of motifs within any signaling pathway in Reactome. As an example, we have annotated the motifs present in the cell cycle (R-HSA-1640170) (Figure 2A, created with Cytoscape (42)), which consists of 610 proteins, 199 of which have motifs annotated in ELM. In Figure 2B, we highlight the 20 proteins involved in the mitotic cell cycle checkpoint (R-HSA-69618), almost all of which have multiple SLiMs (Figure 2A). Degradation motifs recognized by the APC/C complex, an E3 ubiquitin ligase, as well as LIG_MAD2 motifs are prominent in these checkpoint proteins. Many proteins involved in the cell cycle contain one or more functionally important linear motifs and combining SLiM annotation with pathway information will help unravel the roles SLiMs play in the cell.

## THE KINOME IN ELM

In this release, we report an expansion of the portion of the human kinome annotated in ELM, including new motif entries for CDKs (not discussed in this article), MAPKs and Plks.

**Figure 3.**
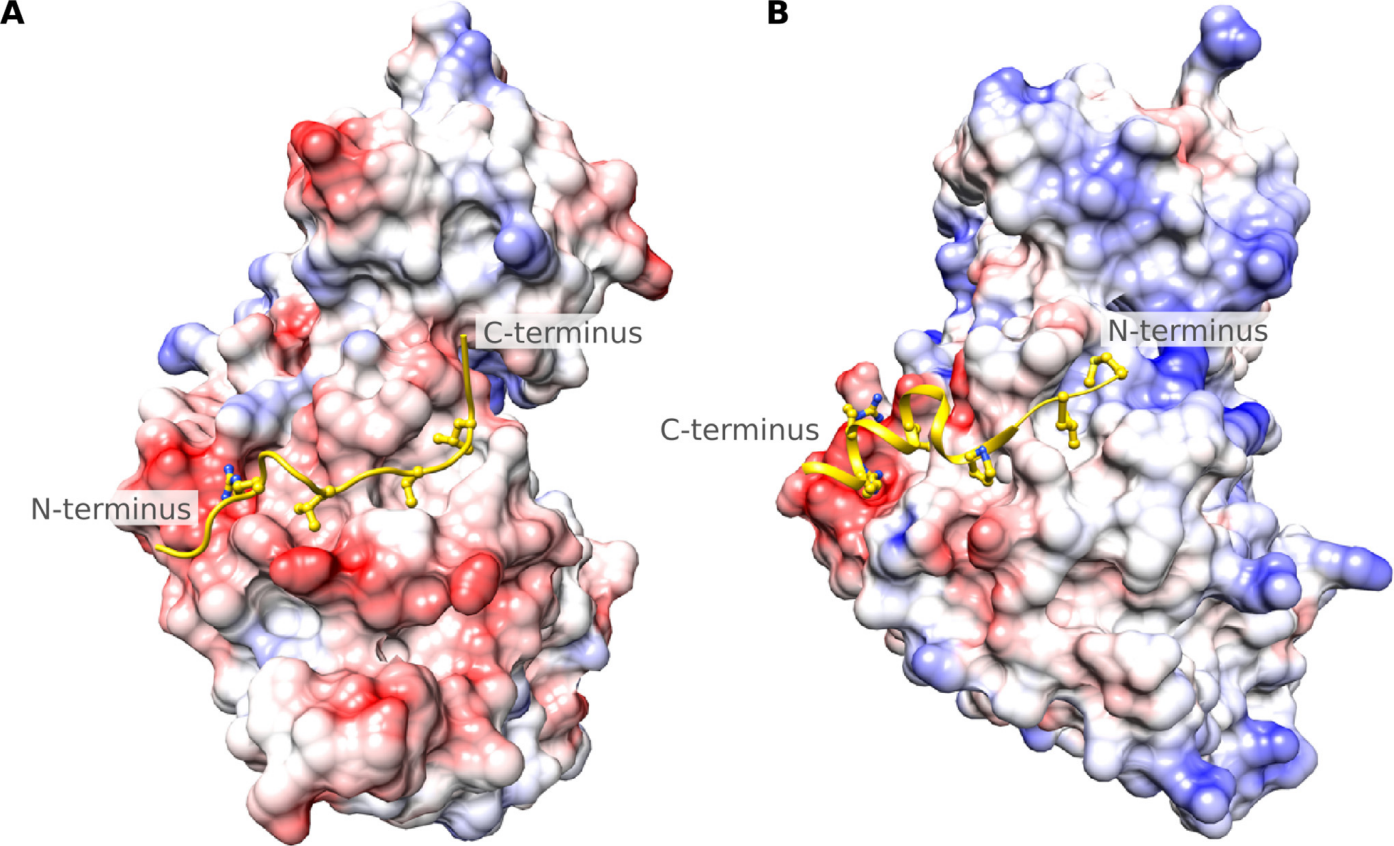
Surface representation showing two MAPK docking motifs bound to the MAPK docking groove. Negative charges and positive charges are shown in red and blue, respectively, on MAPK and the docking motif is rendered in yellow. (**A**) The N- to C-terminal orientation of the MAPK docking motif shown for the DOC_MAPK_NFAT4_5 motif (with regular expression: [RK][∧P][∧P][LIM].L.[LIVMF]). Here, charged amino acids [RK] are followed by hydrophobic residues (PDB:2XS0; (43)). (**B**) The reverse MAPK docking motif shown for the DOC_MAPK_RevD_3 motif (with regular expression: [LIVMPFA].[LIV].1,2[LIVMP].4,6[LIV]..[RK][RK]), where the N-terminus has hydrophobic amino acids followed by charged residues (PDB:2Y9Q; (43)). Figures produced using Chimera (46).

MAPKs form an important part of conserved signaling pathways involved in processes such as cell division, differentiation, growth and apoptosis (43–45). MAPKs are serine/threonine kinases that recognize substrates by the [ST]P motif, and for specificity rely on additional motifs (for example D-motifs) to bring the kinase and its substrate close together for phosphorylation. These motifs harbor one or two basic residues, a variable linker segment and usually three hydrophobic amino acids. Interestingly, the motif orientation can be from the N- to C-terminus where charged residues are followed by linker and hydrophobic residues (for example DOC_MAPK_NFAT4_5, Figure 3A, produced using Chimera (46)) or C- to N-terminus, where hydrophobic residues precede the charged amino acids (e.g. Figure 3B).

Plks are central to the cell cycle and are often found restricted to cellular locations involved in mitosis (such as centrosomes, kinetochores and the spindle) (47). Humans have four functional Plks. The C-terminal parts of Plks 1–3 have two polo-box domains that help target and recruit the kinase substrates by recognizing the short consensus sequence (S[ST]) which, when phosphorylated on the second residue, acts as a docking/activation site (48,49). Specificity is conferred by the Plk’s specific target motif: Plk1 requires an Asp or Glu two positions before the phosphosite, Plks 2 and 3 require an Asp or Glu either two positions before or after the phosphosite and Plk4 has a varied motif requirement where hydrophobic residues are strongly favored after the phosphosite consensus sequence.

## CONCLUSION AND FUTURE DIRECTIONS

Every year ELM continues to grow in terms of new content and connectivity to other resources. As more content is added to ELM we also expect to characterize more and improve existing motif classes. Each addition to the database will allow researchers to uncover new biological insights involving motifs in protein–protein interactions, pathways and networks as well as better understanding the roles of SLiMs in disease and pathogenicity. One of the important aspects of this work will be not only to add new content to the database, but also to review and update the existing content with new discoveries from the scientific literature. We will also continue integrating ELM with existing and emerging bioinformatics resources for SLiM research and protein biology. In parallel we will further develop the ELM API to facilitate the integration of ELM with other bioinformatics tools and resources. We expect that ELM will continue to be a useful and unique resource for SLiM research and the life science community. Users are also encouraged to visit the ‘external links’ page (http://elm.eu.org/infos/links.html) which lists many other useful tools and databases for SLiM research such as QSLiMFinder (for motif discovery (50)) and ProViz (for motif exploration (24)). We also welcome any feedback you can give us that can help us improve ELM.
